# Novel derivative of aminobenzenesulfonamide (3c) induces apoptosis in colorectal cancer cells through ROS generation and inhibits cell migration

**DOI:** 10.1186/s12885-016-3005-7

**Published:** 2017-01-03

**Authors:** Khayal Al-Khayal, Ahmed Alafeefy, Mansoor-Ali Vaali-Mohammed, Amer Mahmood, Ahmed Zubaidi, Omar Al-Obeed, Zahid Khan, Maha Abdulla, Rehan Ahmad

**Affiliations:** 1 Colorectal Research Center, Department of Surgery, King Khalid University Hospital College of Medicine, King Saud University, PO BOX 7805 (37), Riyadh, Saudi Arabia; 2 Department of Chemistry, Kulliyyah of Science, International Islamic University, P.O. Box 141, 25710 Kuantan, Malaysia; 3 Stem Cell Unit, Department of Anatomy, King Khalid University Hospital College of Medicine, King Saud University, Riyadh, Saudi Arabia; 4 Genome Research Chair, Department of Biochemistry, College of Science, King Saud University, Riyadh, Saudi Arabia

**Keywords:** Colorectal cancer, ROS, NAC, Apoptosis, Cell migration

## Abstract

**Background:**

Colorectal cancer (CRC) is the 3^rd^ most common type of cancer worldwide. New anti-cancer agents are needed for treating late stage colorectal cancer as most of the deaths occur due to cancer metastasis. A recently developed compound, 3c has shown to have potent antitumor effect; however the mechanism underlying the antitumor effect remains unknown.

**Methods:**

3c-induced inhibition of proliferation was measured in the absence and presence NAC using MTT in HT-29 and SW620 cells and xCELLigence RTCA DP instrument. 3c-induced apoptotic studies were performed using flow cytometry. 3c-induced redox alterations were measured by ROS production using fluorescence plate reader and flow cytometry and mitochondrial membrane potential by flow cytometry; NADPH and GSH levels were determined by colorimetric assays. Bcl2 family protein expression and cytochrome c release and PARP activation was done by western blotting. Caspase activation was measured by ELISA. Cell migration assay was done using the real time xCELLigence RTCA DP system in SW620 cells and wound healing assay in HT-29.

**Results:**

Many anticancer therapeutics exert their effects by inducing reactive oxygen species (ROS). In this study, we demonstrate that 3c-induced inhibition of cell proliferation is reversed by the antioxidant, N-acetylcysteine, suggesting that 3c acts via increased production of ROS in HT-29 cells. This was confirmed by the direct measurement of ROS in 3c-treated colorectal cancer cells. Additionally, treatment with 3c resulted in decreased NADPH and glutathione levels in HT-29 cells. Further, investigation of the apoptotic pathway showed increased release of cytochrome c resulting in the activation of caspase-9, which in turn activated caspase-3 and −6. 3c also (i) increased p53 and Bax expression, (ii) decreased Bcl2 and BclxL expression and (iii) induced PARP cleavage in human colorectal cancer cells. Confirming our observations, NAC significantly inhibited induction of apoptosis, ROS production, cytochrome c release and PARP cleavage. The results further demonstrate that 3c inhibits cell migration by modulating EMT markers and inhibiting TGFβ-induced phosphorylation of Smad2 and Samd3.

**Conclusions:**

Our findings thus demonstrate that 3c disrupts redox balance in colorectal cancer cells and support the notion that this agent may be effective for the treatment of colorectal cancer.

**Electronic supplementary material:**

The online version of this article (doi:10.1186/s12885-016-3005-7) contains supplementary material, which is available to authorized users.

## Background

Colorectal cancer (CRC) is the second leading cause of cancer-related deaths in the US and is associated with high mortality. CRC is the 3^rd^ most common cause of cancer globally [1]. The basis for the high mortality in patients with colorectal cancer is the formation of distant metastasis. Colorectal cancer patients diagnosed at early stage have a 5 year-survival rate of about 90%, which decreases to 65% with lymph node metastasis and to <10% with distant metastasis [2]. CRC is a heterogenous disease with progressive accumulation of genetic and epigenetic alterations [3]. Oncogene activation and loss of tumor suppressor genes are crucial for transformation from normal cells to cancer cells [4].

Oxidative stress has been shown to be involved in diverse physiological and certain pathological conditions, such as cancer [5]. Reactive oxygen species (ROS) are produced in a cell as a result of normal metabolic processes, as well as xenobiotic exposures. The concentration of ROS determines its beneficial or harmful effects for the cells and tissues [6]. Owing to their basal higher ROS levels as compared to normal cells, cancer cells are more susceptible when encountering additional ROS insults induced by anticancer agents [7]. Excess levels of ROS can trigger cell death by activating pathways leading to apoptosis, necrosis and autophagy [8, 9]. Various studies have reported activation of p38 MAPK and JNK pathways in ROS mediated apoptotic cell death [10–12]. Moreover, Akt has been shown to be regulated by ROS [13, 14].

Elevated levels of ROS promote genotoxic damage and thereby cancer progression by amplifying genomic instability and also by stimulating tumor promoting signaling pathways [15]. Consequently, oxidative stress provides a growth advantage to transformed cells by activating signaling pathways that stimulate proliferation and maintenance of cancer cells. However, excessive levels of ROS can have deleterious effect on cancer cells and can readily induce cell cycle arrest and apoptosis. As a result, selectively targeting cancer cells by modulating ROS levels has been proposed as an effective therapeutic strategy. Several studies showed effect of anticancer agents that increases the ROS levels to efficiently kill cancer cells [16, 17]. Abnormal increases in the reactive oxygen species during the oncogenic transformation process render cancer cells sensitive to oxidative stress inducing agents [18]. These agents by further elevating ROS levels beyond the antioxidant capacity of the malignant cells induce apoptosis. However, these ROS inducers have no or minimal effects on normal cells due to their low production of ROS and high antioxidant capacity. Thus, oxidative stress in cancer cells has the potential to be exploited in the development of novel and selective anticancer therapeutics. Anti-cancer drugs being used for the treatment of colorectal cancer include 5-flurouracil, oxaliplatin and irinotecan [4]. The treatment involve conventional therapy which include surgical resection, chemotherapy and radiation, all of them are often inadequate in treating colorectal cancer. Therefore, new treatment options are urgently needed. Despite the discovery of novel targeted agents and use of different combination therapeutics, no treatment regimens are available for treating colorectal cancer patients with distant metastasis.

Recently, we discovered a novel derivative of aminobenzenesulfonamide (2-substituted-quinazolin-4-yl-aminobenzenesulfonamide), designated 3c, as a potential anti-tumor agent [19]. The aim of the present study was to investigate the mechanism of 3c-induced inhibition of cellular proliferation. This study demonstrated that inhibitor 3c induces apoptosis mediated by increased production of reactive oxygen species in colorectal cancer cells. Furthermore, 3c reduces NADPH and GSH levels along with up-regulation of cytochrome c, cleaved PARP and activation of caspases. In addition, the small molecule 3c inhibited the cell migration of colorectal cancer cells. These findings identify ROS induction as the primary mechanism of action of compound 3c and support the development and use of oxidative stress inducers as anticancer agents.

## Methods

### Cell culture

Human HT-29 and SW620 colorectal cancer cells were obtained from ATCC (Manassas VA) and grown in RPMI (Invitrogen) containing 10% heat-inactivated fetal bovine serum, 100 μg/ml streptomycin, 100 units/ ml penicillin and 2 mmol/l L-glutamine. In certain experiment TGFβ stimulation was done at 10 ng/ml.

### Cell viability assay

Cell viability was determined using MTT [19]. Briefly, after culturing cells in 96 well plate for 24 h, they were treated with 3c (5 μM) for 24 h. Freshly prepared 10 μL of MTT 3-(4, 5-dimethylthiazolyl-2)-2, 5-diphenyltetrazolium bromide) (5 mM) solutions were added to the cells and was further incubated for 2 h at 37 °C in 5% CO2. 100 μL of dimethyl sulfoxide (DMSO) were added in each well to dissolve the crystal of formazan, which formed in the reaction of MTT at the time of incubation. The crystals were dissolved through pipetting carefully. The absorbance of the product was measured at 540 nm using a microplate reader. The experiments were performed in triplicates for each condition. The graph illustrates the mean and standard deviation (SD) values of three independent experiments.

### Cytotoxicity assay using xCELLigence system

Optimal seeding concentration for proliferation of HT-29 was determined. HT-29 cells (5000 cell in 150 μL medium/well) were seeded in 16 well plates (E-plate 16 ACEA Biosciences Inc, San Diego USA) following the xCELLigence Real Time Cell Analyzer (RTCA) DP instrument manual as provided by the manufacturer. After 24 h 3c (5 μM) was added and the experiment was allowed to run for 3–4 days. Baseline cell index were calculated for at least two measurements from three replicate experiments. Appropriate wells were pre-treated with NAC (5 mM) for 1 h and then compound 3c (5 μM) was added. Cell proliferation was monitored for another 72 h.

### Apoptosis

Cells treated with compound 3c in the presence and absence of NAC was incubated with propidium iodide (PI)/annexin V–FITC (BD BioSciences) for 15 min at room temperature and then analyzed by flow cytometry on FACSCalibur (BD Biosciences).

### Western blotting

Whole cell lysates were prepares using RIPA lysis buffer as described [20]. Total protein concentration was determined using Bradford Protein reagent (Bio-Rad). Soluble proteins were loaded on precast TGX gels and were analyzed by immunoblotting with anti-cytochrome c (1:200 Abcam), anti-PARP (1:200 BioVision), anti-Bax, anti-Bcl2, anti-BclxL, anti-Cyclin D1 (Dilution 1:1000; Santa Cruz Biotechnology) and anti-β-actin (1:10,000 Sigma). Reactivity was detected with horseradish peroxidase-conjugated secondary antibodies and chemiluminescence by Clarity Western ECL Substrate (Bio-Rad). Membrane was developed using C-Digit Blot Scanner (LI-COR, Hamburg Germany).

### Cytochrome C measurement

Briefly, cells were treated with 3c for 18 h and harvested cells were homogenized in 1X cytosolic extraction buffer by 30 strokes of a Dounce homogenizer using mitochondria isolation Kit (Abcam ab65311). The homogenate was centrifuged at 600 × *g* for 5 min, and the resulting supernatant was centrifuged at 10,000 × *g* for 10 min. The mitochondrial pellet was washed with the buffer and resuspended in mitochondrial extraction buffer. Mitochondria and cytosolic extracts were immunoblotted for cytochrome c.

### Reactive Oxygen Species (ROS) measurement

Intracellular ROS accumulation was monitored in HT-29 cells by adding the H_2_-DCFDA [21]. In brief, 5000 cells/well were seeded with phenol free DMEM in a 96-well microplate. The cells were treated with 3c for 18 h. DCFDA was added to the wells at 5 μM for 30 min. Increases in fluorescence were measured at excitation and emission wavelengths of 485 and 535 nm, respectively.

### ROS measurement by flow cytometry

Cells were pretreated with compound 3c (5 μM) for different time points. Cells were then treated with c-H_2_DCFDA (5uM) for 20 min at 37C to assess hydrogen peroxide (H2O2)-mediated oxidation to fluorescent compound DCF [22]. Fluorescence of oxidized DCF was measured using flow cytometry (BD FACS Calibur) at excitation wavelength of 480 nm and emission wavelength of 525 nm.

### Measurement of mitochondrial membrane potential

Cells were treated with 3c (5uM) for different time points then cells were incubated with rhodamine 123 (25 ng/ml) (Molecular Probes) in PBS for 20 min at 37C. Rhodamine 123 positive populations were monitored using flow cytometry [22].

### GSH measurement

The levels of GSH in the cells were determined according to the method based on the formation of 2-nitro-5-tiobenzoic acid from DTNB in the presence of GSH [21]. In brief, 25 μl of trichloroacetic acid (15%) was added to 50 μl of the homogenate, followed by centrifugation at 13,000 x *g* for 5 min at 4 °C. A supernatant aliquot (50 μl) was mixed with 50 μl of 3.4 mM ethylenediaminetetraacetic acid (EDTA) dissolved in PBS, 1 ml of PBS, and 250 μl of DTNB in PBS (20 mg/ml). The absorbance was measured at 412 nm after 15 min and compared to a standard curve of GSH (0.01–0.5 mM).

### Determination of NADPH levels

Intracellular NADPH concentrations were measured using the NADP/NADPH Assay Kit as per the manufacturer’s instructions (BioVision, Milpitas, CA USA).

### Caspase activity assay

Caspase activity assay was determined using Caspase Colorimetric Protease Assay Sample Kit for measuring Caspase-2, −3, −6, −8, −9 (Invitrogen KHZ1001) at 400 nm on microplate reader.

### Cell migration assay

For monitoring of cell migration in real-time the xCELLigence Real Time Cell Analyzer Dual Plate (RTCA-DP) instrument was used according to the manufacturer’s recommendations (Acea Biosciences Inc USA). The impedance is expressed as a dimensionless parameter, termed cell index, and is directly proportional to the area covered by cells. For detection of cellular migration, electrical impedance changes are measured at a gold microelectrode plated on the bottom of a membrane separating the upper and lower chambers. The SW620 cell line was treated without and with 3c and subjected to serum starvation 12 h before the start of measurement. For cell migration assays, 12 x 10^3^ cells in RPMI-1640 were seeded per well of a 16-well CIM plate, and the lower chamber was loaded with RPMI-1640 supplemented with 10% FCS. Cell index values were monitored every 5 min for 150 h. At least three independent experiments were performed for monitoring cell migration each carried out in triplicates. Cell index values were calculated and plotted using the RTCA software 1.2.1 of the RTCA xCELLigence system. For cell migration, the cell index curves were monitored for 150 h.

### Wound healing assay

Wound healing assay was performed as described by Li et al. [23]. HT-29 cells were seeded in at 1x10^6^ cells/well in duplication. Cells were allowed to grow to near confluency for three days and scratched a wound through the center of the well. Washed with PBS three times, DMSO and 3c compound was added in duplicate. After taking pictures under microscope (10X), plates were transferred to CO2 incubator at 37 °C and incubated for 48 h. After final incubation, pictures were again taken under microscope.

## Results

### 3c induced inhibition of cell proliferation is reversed by NAC

**Fig. 1:**
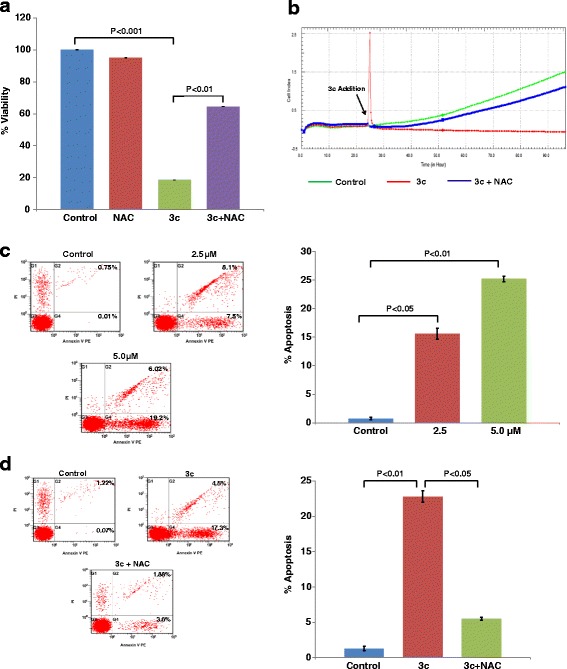
NAC reverses the cytotoxicity induced by 3c. **a**. HT-29 cells were treated with 3c (5 μM) in the absence and presence of NAC (5 mM), Cell viability was measured by MTT assay. The results are expressed as mean of 3 independent determinations (mean ± SD). **b**. 5 x 10^3^ cells were seeded in E-plate 16, after overnight incubation, cells were pre-treated with NAC followed by treatment with 3c. Real-time proliferation monitoring of HT-29 cells was performed by measuring cell index to evaluate the cytotoxic effect of 3c using xCELLigence RTCA-DP system. **c**-**d**. HT-29 cells were treated with various concentration of 3c for 24 h. 0.5% DMSO was used as a control. Cells were processed for flow cytometry using Annexin V/PI staining. The percentage of Annexin V+ population indicates apoptosis induction. Results shown are representative of 3 independent experiments. In some cases where indicated cells were pre-treated with NAC for 1 h followed by 3c treatment

Compound 3c is a novel derivative of aminobenezenesulfonamide that has been shown to inhibit cellular proliferation in colorectal cancer cells [19]. ROS is a major signaling molecule mediating the effect of anticancer therapeutics. Hence, in this study, we investigated whether 3c induced inhibition of cell proliferation is mediated by reactive oxygen species (ROS). Compound 3c had significant cytotoxic effects on HT-29 colorectal cancer cells that showed a marked reduction in viability to 18% following 24 h treatment with the drug. Noticeably, the antioxidant N-acetylcysteine (NAC) was found to significantly reverse the effect of 3c (Fig. 1a). Similar result was obtained in another colorectal cancer cells, SW620 (Additional file 1: Figure S1). This finding was confirmed by using the xCELLigence real time cell proliferation system, whereby NAC as observed earlier was found to inhibit the cytotoxicity induced by 3c in colon cancer cells (Fig. 1b). To further investigate whether 3c mediated cell death is caused by apoptosis or necrosis, we performed flow cytometry on HT-29 colorectal cancer cells. As shown in Fig. 1c, 3c treatment significantly induced early apoptosis in a dose dependent manner (Fig. 1c). Pretreatment of the cancer cells with NAC markedly blocked the induction of apoptosis supporting the role of oxidative stress (Fig. 1d). These findings thus indicate that 3c induced inhibition of cell proliferation is mediated by ROS. To compare the effect of 3c with standard drug, doxorubicin is known to inhibit HT-29 cell proliferation. Herein doxorubicin was found to decrease cell viability in dose dependent manner. 3c significantly potentiated Dox-mediated inhibition of cell viability (Additional file 1: Figure S2). Of note 3c has no cytotoxic effect on human mammary epithelial cells MCF10A (Additional file 1: Figure S3). These findings confirmed that 3c exerts cytotoxic effect in cancer cells having no or minimal effect on normal cells.

### 3c modulates redox balance

**Fig. 2:**
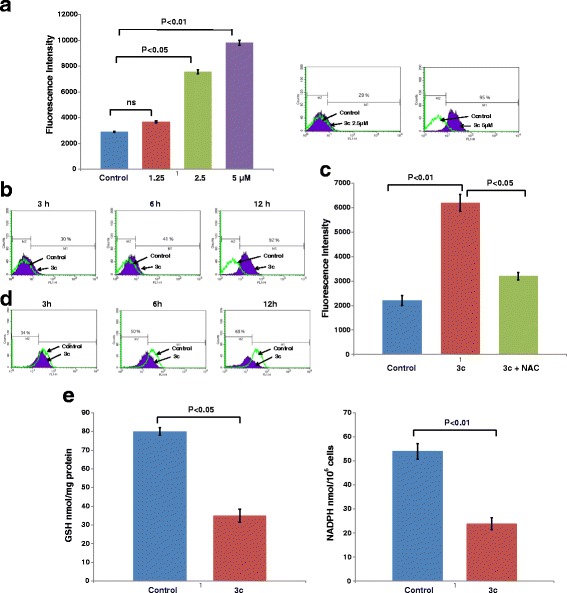
Compound 3c modulates Redox balance. **a**. 3c treated HT-29 cells were incubated with c-H2-DCFDA for 30 min. The fluorescence of the oxidized DCF was measured using Fluorescence plate reader (left). HT-29 cells treated with 3c were incubated with c-H_2_DCFDA for 15 min. Fluorescence of oxidized DCF was measured by flow cytometry (right) **b**. HT-29 cells were treated with 3c for different time points and then incubated with c-H_2_DCFDA for 15 min. Fluorescence of oxidized DCF was determined using flow cytometry. **c**. NAC pre-treated cells were incubated with 3c for 24 h followed by incubation with c-H2-DCFDA for 30 min. Fluorescence was measured and results expressed as mean ± SD of 3 determination. **d**. HT-29 cells were treated with 3c for different time points and incubated with rhodamine 123 and analyzed by flow cytometry. **e**. Cells were analyzed for GSH levels using DTNB and presented as nmol/mg protein (left). 3c treated cells were analyzed for NADPH levels (right). The results are expressed as the NADPH levels (mean ± SD of 3 determinations)

To assess whether compound 3c induces oxidative stress, we utilized the property of 2',7' –dichlorofluorescin diacetate (DCFDA) fluorogenic dye which after oxidization emits green fluorescence. Treatment of HT-29 cells with 3c inhibitor was associated with increases in ROS as compared to the untreated control (Fig. 2a Left). Production of ROS by 3c was again confirmed using flow cytometry (Fig. 2a Right). 3c was found to enhance ROS production in metastatic colorectal cancer cell line, SW620 as well (Additional file 1: Figure S4). Treatment of cells with 3c was associated with increases in hydrogen peroxide in a time dependent manner that reached maximal levels at 12 h (Fig. 2b). The induction of ROS by 3c was reversed by NAC (Fig. 2c). A major source of ROS is produced by the mitochondria electron transport chain. Moreover, oxidative stress inhibits the mitochondrial membrane potential. In this regard, rhodamine 123 staining of HT-29 cells demonstrated that 3c decreases the mitochondrial membrane potential in a time dependent manner (Fig. 2d). Similarly 3c also inhibited mitochondrial membrane potential in SW620 cells (Additional file 1: Figure S5). Treatment with 3c was also associated with reduction in GSH and NADPH levels (Fig. 2e). In summary, 3c results in increased levels of ROS and decreased NADPH and GSH. These findings thus indicate that 3c disrupts redox balance in colorectal cancer cells with increases in hydrogen peroxide and decreases in NADPH and GSH.

### 3c-induces modulation of Bcl2 family protein and inhibits Cyclin D1

**Fig. 3:**
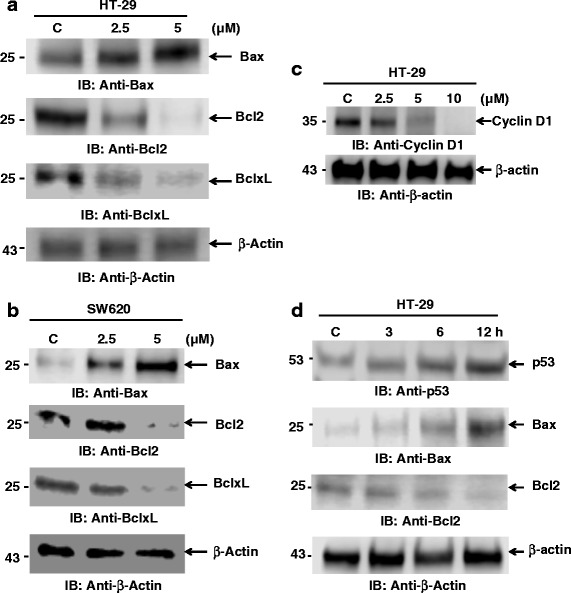
3c alters Bcl2 family proteins and inhibits Cyclin D1. **a**. HT-29 cells were treated with different concentration of 3c for 24 h. Cell lysates were immunoblotted with the indicated antibodies. **b**. SW620 cells were treated with various concentration of 3c for 24 h. Cell lysates were immunoblotted with indicated antibodies. **c**. Total cell lysate from HT-29 cells treated with different concentration of 3c, were immunoblotted with the indicated antibodies. **d**. Cells were treated with 3c for different time points, total cell lysates were immunoblotted with the indicated antibodies

The interplay between the pro-apoptotic Bax and anti-apoptotic Bcl2 proteins results in the release of cytochrome c from mitochondria, which leads to caspase activation and subsequent apoptosis. Incubation of cells with 3c increased expression of Bax (Fig. 3a). Additionally, 3c treatment inhibited the expression of the anti-apoptotic Bcl2 and BclxL proteins. Similar results were obtained from studies with the metastatic colorectal cancer cell line SW620 (Fig. 3b). Cyclin D1 is a major proliferation gene known to regulate cell cycle progression. 3c resulted in the inhibition of cyclin D1 expression in dose dependent manner (Fig. 3c). To understand the mechanism for Bax induction, p53 is a well-known tumor suppressor involved in apoptosis, cell cycle regulation and DNA repair. Bax and Bcl2 are transcriptional targets of p53 [24]. p53 has been shown to induce Bax oligomerization and cytochrome c release from mitochondria. Notably, treatment of cells with 3c increased p53 expression in a time dependent manner (Fig. 3d). Bax expression was also found to be increased and Bcl2 expression was decreased. These findings indicate that 3c modulates Bcl family proteins by increasing Bax expression and inhibiting antiapoptotic proteins favoring the balance towards apoptosis along with inhibiting cell cycle regulator cyclin D1.

### 3c activates Cytochrome c release, PARP cleavage and Caspase cascade

**Fig. 4:**
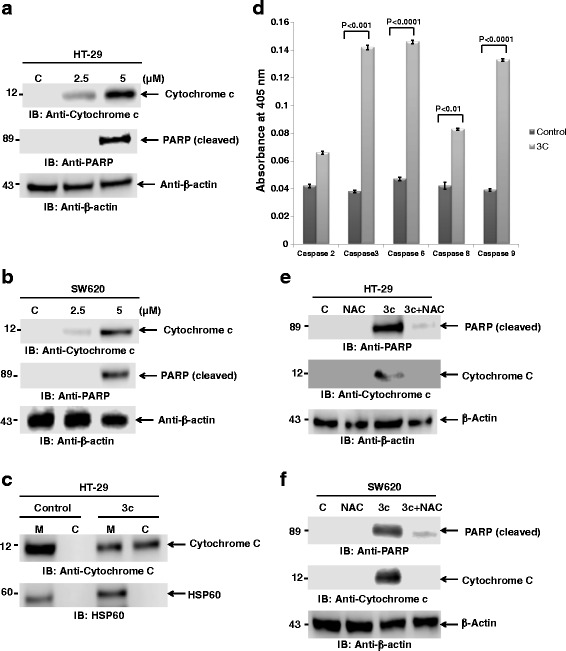
3c activates cytochrome c release, PARP and caspase cascade. **a**. HT-29 cytosolic extracts were immunoblotted with the indicated antibodies. **b**. SW620 cytosolic extracts were immunoblotted with the indicated antibodies. **c**. HT-29 cells were treated with 3c, mitochondrial and cytosolic extracts were isolated and immunoblotted with the indicated antibodies. **d**. Cells were treated with 3c for 24 h, cytosolic extracts were isolated and ELISA was performed for caspases activity. **e**. HT-29 cells were pre-treated with NAC (5 mM) for 1 h followed by 3c treatment. Cytosolic extracts were immunoblotted with the indicated antibodies. **f**. SW620 cells were pre-treated with NAC (5 mM) for 1 h followed by 3c treatment. Cytosolic extracts were immunoblotted with the indicated antibodies

There are two major classes of initiator (i.e., caspase-9) and effector caspases (i.e., Caspases-3 and −6). Cytochrome c release from mitochondria binds with APAF1 and ATP leading to the activation of the initiator caspase-9, which in turn activates the effector caspase-3 and 6. Involvement of cytochrome c release from mitochondria is an indicator of activation of the intrinsic apoptotic pathway. Treatment with 3c induces cytochrome c in human colorectal cancer cells (Fig. 4a). Similar results were also obtained in SW620 colorectal cancer cells (Fig. 4b). Furthermore, 3c induces cytochrome c release from mitochondria into the cytosol (Fig. 4c). To investigate which caspase pathway is activated by 3c for induction of apoptosis, ELISA was performed with various caspase substrates. HT-29 cells treated with 3c exhibited enhanced activation of caspase-9; thereby activating effector caspase-3 and 6 (Fig. 4d). A modest increase in caspase-8 activity was also observed. This result indicates that 3c induced apoptosis is mediated predominantly by the intrinsic apoptosis pathway and to a limited extent the extrinsic pathway. PARP (Poly-ADP-ribose polymerase) is a family of proteins known to be involved in various cellular processes like DNA repair and programmed cell death. PARP can be cleaved by many caspases and is the main cleavage target of caspase-3. Our results further demonstrate that 3c induces the cleavage of PARP as shown by an increase in cleaved PARP in HT-29 cells (Fig. 4a) and in SW620 cells (Fig. 4b). These results suggest that compound 3c induces apoptosis by release of cytochrome c, caspase activation and cleavage of PARP.

To determine whether the 3c-induced apoptotic markers observed in human colon cancer cells is mediated by elevated ROS levels, we examined the activation of cleaved PARP and upregulation of cytochrome c by 3c in cells pretreated with NAC. HT-29 cells were pretreated with or without 5 mM NAC for 1 h and were then subjected to 3c treatment for an additional 24 h. As expected, NAC abolished the activation of PARP by 3c (Fig. 4e). Similarly NAC was found to inhibit the release of cytochrome c into the cytosol (Fig. 4e). Similar results were obtained with SW620 cells (Fig. 4f).

### 3c inhibited cell migration

**Fig. 5:**
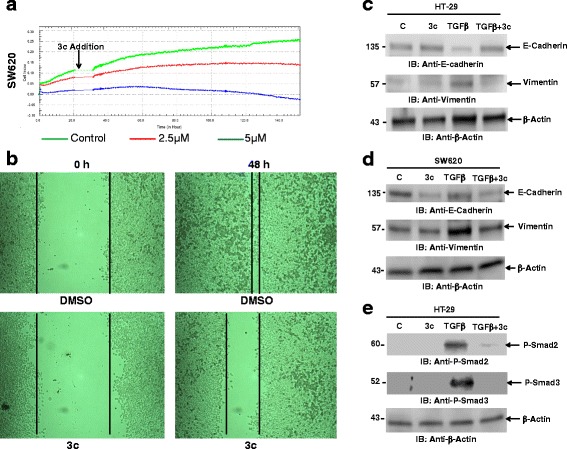
Compound 3c inhibits migration of cancer cells. **a**. SW620 cells were seeded in the designated upper chamber and real time migrations of cells were monitored using xCELLigence RTCA-DP system. **b**. HT-29 cells were seeded into a 6-well plate and allowed to grow to 90% confluency in complete media. Cell monolayers were wounded by tips (1 mm) and washed with PBS. Media replaced with fresh media with either DMSO or 3c and put back into the incubator for 48 h. Cells were monitored by microscope and digital images were captured. All the experiments were repeated three times and the representative images were shown. **c**: HT-29; **d**: SW620; **e**: HT-29 cells were pretreated with 3c (5 μM) and stimulated with TGFβ, total cell lysates were immunoblotted with the indicated antibodies

The potential of cancer cells to migrate is of importance for cancer metastasis. To assess the effect of 3c on cell migration, we employed the SW620 colorectal cancer cells which are derived from late stage metastatic cancer. We analyzed the ability of these cells to migrate through porous membrane using xCELLigence system for real time data recording of cell migration. In the cell migration assay of the xCELLigence system, cells with higher potential to migrate attach to the bottom side of the membrane in the top chamber. This attachment of migrated cells increases the electrical impedance. 3c induced a delay of signals as compared to control (Fig. 5a). This decrease in cell migration was also dose dependent. Integrating the area under the signal curves of three independent experiments showed a significant reduction in cell migration by 3c as compared to untreated controls. These results confirmed that 3c induces apoptosis and also has anti-migration effects. To further explore the effect of 3c on cell migration using a different approach, the wound healing assay was used to study HT-29 colorectal cancer cells. Cell migration was evaluated by monitoring the closing of an applied scratch on a cell monolayer. As shown, wound healing was significantly impaired in 3c treated HT-29 cells as compared to control cells (Fig. 5b), suggesting that 3c inhibits cell motility and thereby cell migration in colorectal cancer cells. To further elucidate the mechanism by which 3c inhibits the cell migration, the epithelial to mesenchymal transition (EMT) is an essential step towards tumor invasion, migration and metastasis [25]. During EMT, expression of E-cadherin and vimentin is decreased and increased respectively. The TGFβ-Smad pathway plays an important role in cell proliferation, differentiation, adhesion, EMT, migration and angiogenesis [25]. TGFβ treatment decreased E-cadherin expression in HT-29 cells. 3c treatment of cells reversed the TGFβ-induced decrease in E-cadherin expression (Fig. 5c). 3c also inhibited Vimentin expression induced by TGFβ treatment. SW620 cells are partly mesenchymal and known to express both E-cadherin and Vimentin. 3c treatment alone inhibited E-cadherin expression without altering Vimentin expression though 3c inhibits TGFβ-induced Vimentin expression in SW620 cells (Fig. 5d). TGFβ phosphorylates Smad2 and Smad3 and forms a heterodimeric complex with Smad4 and translocate into the nucleus to regulate target genes. 3c inhibited TGFβ-induced phosphorylation of Smad2 and Smad3 (Fig. 5e). Thus, these findings indicate that 3c inhibits TGFβ-induced Smad phosphorylation and EMT markers as mechanism for cell migration inhibition.

## Discussion

We recently discovered a novel quinazolin-4-sulphomide derivative (3c) as a potential anti-tumor agent [19]. However the mechanism by which this compound acts remains unknown. In this study we shed new light on the mechanism by which 3c exerts its anti-cancer activity. Our results show that 3c induces apoptosis in human colorectal cancer cells by increased ROS production. ROS, which includes hydrogen peroxide, hydroxyl radical, and superoxides, are chemically active prooxidant molecules generated by incomplete reduction of oxygen [26]. Reactive oxygen species are involved in a variety of cellular physiology and pathology [27]. Anti-cancer agents-induced ROS production that mediates the induction of apoptosis has been exploited in designing effective strategies for cancer therapeutics [26]. Many studies investigated the effects of both natural and synthetic anti-cancer agents which modulate ROS in colorectal cancer [28, 29]. 3c was found to increase ROS in colorectal cancer cells and was blocked by NAC. Our results support a model in which 3c-induced ROS production stimulates the intrinsic mitochondrial apoptotic pathway by decreasing outer mitochondrial membrane potential and thereby releasing cytochrome c that further activates caspase dependent signaling [30]. ROS are well known mediators of the intracellular signaling cascade known to play an important role in cancer drug discovery [31, 32]. They play critical role in the regulation of diverse functions, such as proliferation, apoptosis and transformation. When ROS levels reach a threshold point beyond the capacity of cellular antioxidant machinery it leads to oxidative stress, which in turn modulate mitochondrial membrane potential [33]. Several studies suggest that cancer cells are under increased oxidative stress associated with oncogenic transformation and increased ROS generation possibly due to its higher metabolic activity [34, 35]. A further increase in the ROS levels can make these malignant cells more vulnerable to cell death relative to untransformed cells. Thus, in cancer therapeutics, ROS signaling can be exploited to develop novel drugs to inhibit or kill cancer cells through its specific ROS signaling mechanism. Our newly developed compound 3c utilizes this mechanism in killing tumor cells. 3c treatment resulted in high level of ROS in HT-29 cells whereas NAC combined with 3c reduced ROS production and subsequently inhibited apoptosis. The molecular mechanism by which 3c induces elevated ROS production is presently not understood. Previous findings have reported that sulphonamide derivatives inhibit carbonic anhydrases activity [36–38]. 3c is known to inhibit the carbonic anhydrase IX and XII expression [19]. Some carbonic anhydrases have been reported to possess antioxidant property. Specifically, carbonic anhydrase III (CA III) and VII (CA VII) are known to have antioxidant characteristics [39–42]. This compound 3c may inhibit CA III and/ or CAVII expression and thereby increases ROS generation. Other mechanism may be involved as well like acting on electron transport chain or by abrogating key antioxidant systems in cells like depleting glutathione pool and/ or inhibiting superoxide dismutase (SOD) [43]. There are some anticancer agents for example elesclomol which is in clinical trial exerts its effect by inducing ROS but mechanism for ROS generation is not known [44]. Oxidative stress leads to decreased mitochondrial membrane potential. In this study 3c was also found to decrease mitochondrial membrane potential. We further showed that 3c induced PARP cleavage is blocked in the presence of NAC. Cytochrome c release induced by 3c was also inhibited by NAC, proving that indeed 3c induced apoptotic markers are ROS dependent. NADPH is required for the conversion of oxidized glutathione to reduced glutathione. 3c treatment resulted in decreased levels of NADPH and GSH reducing the cells antioxidation capacity and thus increasing oxidative stress.

We further uncovered the molecular pathway underlying 3c-induced apoptosis through upregulation of pro-apoptotic protein, Bax leading to the cytochrome c release from mitochondria. Cytochrome c release from mitochondria leads to the activation of caspase cascade which is essential in initiating apoptosis by anti-cancer agents [45]. 3c treatment resulted in an increase in caspase-9 and effector caspase-3 and 6 activities. Caspase-9 is an initiator caspase in mitochondria mediated apoptosis pathway [46]. These findings indicate that 3c induces apoptosis through the intrinsic pathway. Certain caspases also target PARP for its cleavage into 24 kDa and 89 kDa fragments, rendering them incapable of DNA repair and leading to cell death. In this study, 3c was found to increase cleaved PARP levels. Cancer cells express series of anti-apoptotic proteins such as Bcl2 and BclxL. Overexpression of these anti-apoptotic proteins inhibits apoptosis and promotes cancer cell survival [47]. The anti-apoptotic Bcl2 protein has been demonstrated to be overexpressed in colorectal cancers [48]. According to Bonnotte et al., Bcl2 mediated apoptosis inhibition restores the tumorigenicity of colon tumors [49]. The reduced expression of Bcl2 and BclxL in our investigation suggests that 3c-induced apoptosis is mediated by an inhibition of these proteins. Zhu and colleagues have shown that induction of Bax expression is essential for death-receptor mediated apoptosis in colon cancer cells [50]. Tumor suppressor p53 is a known regulator of Bcl2 and Bax gene expression [24]. p53 may be involved in the 3c-induced Bax expression and downregulation of Bcl2 expression. 3c was found to increase p53 and Bax expression in a time dependent manner similarly decreasing Bcl2 expression. This finding may provide a mechanism for 3c-induced alterations of Bcl family protein by involving p53 transcription factor. Our observation of elevated Bax expression and an increase in caspase-8 activity in 3c treated colorectal cancer cells may suggest involvement of extrinsic apoptotic pathway as well. However, it is possible that caspase-8 through cleavage of BH3-only protein, BID also activates intrinsic apoptosis pathway [51]. Additionally we observed an important property of 3c to inhibit cancer cell migration that would have a significant clinical benefit in controlling invasion and metastasis. Epithelial to mesenchymal transition (EMT) is essential process for cell invasion and migration [25]. 3c was found to alter the expression of EMT markers like E-Cadherin and Vimentin. TGFβ-smad pathway is known to induce EMT in cancer cells. This compound of interest, 3c inhibits TGFβ-induced smad pathway in colorectal cancer cells. These findings thus indicate that 3c-induced inhibition of cell migration is mediated by TGFβ-smad pathway.

## Conclusions

The present results indicate that compound 3c 1) induces apoptosis by increasing ROS levels, 2) increases p53 expression modulate Bcl2 family proteins expression, 3) leads to PARP cleavage and cytochrome c release 4) reduces NADPH and GSH levels and 5) suppresses cancer cell migration by altering EMT markers and inhibits TGFβ dependent phosphorylation of Smads. These in-vitro results suggest that 3c is an attractive candidate for further investigation as potential anticancer agent. More studies are needed for the combination studies with other known therapeutics and for the in-vivo effect of 3c. A deeper understanding of the molecular mechanism of anti-cancer activity of 3c will allow for precise treatment regimens and combination therapeutics.

## Additional file

Additional file 1: Figure S1. SW620 cells were treated with 3c (5 μM) in the absence and presence of NAC (5 mM), Cell viability was measured by MTT assay. The results are expressed as mean of 3 independent determinations (mean ± SD). **Figure S2.** HT-29 cells were treated with different concentration of Doxorubicin in the absence and presence of 3c (5 μM), Cell viability was measured by MTT assay. The results are expressed as mean of 3 independent determinations (mean ± SD). **Figure S3.** MCF10A cells were treated with different concentration of 3c, Cell viability was measured by MTT assay. The results are expressed as mean of 3 independent determinations (mean ± SD). **Figure S4.** SW620 cells treated with 3c were incubated with c-H_2_DCFDA for 15 min. Fluorescence of oxidized DCF was measured by flow cytometry. **Figure S5.** 3c treated SW620 cells were incubated with rhodamine 123 and analyzed by flow cytometry. (PPTX 115 kb)

## Abbreviations

CRC: Colorectal cancer
DCFDA: 2',7'-dichlorodihydrofluorescein diacetate
DMSO: Dimethyl sulfoxide
DTNB: 5,5-dithio-bis-(2-nitrobenzoic acid)
EDTA: Ethylenediaminetetraacetic acid
GSH: Glutathione
MTT: 3-(4, 5-dimethylthiazolyl-2)-2, 5diphenyltetrazolium bromide
NAC: N-acetylcysteine
NADPH: Nicotinamide adenine dinucleotide phosphate
PI: Propidium iodide
ROS: Reactive oxygen species.
