# Supplementary material for: Novel derivative of aminobenzenesulfonamide (3c) induces apoptosis in colorectal cancer cells through ROS generation and inhibits cell migration
Source: BMC Cancer. 2017 Jan 3;17:4. doi: 10.1186/s12885-016-3005-7 (PMC5210304; doi:10.1186/s12885-016-3005-7)
Supplement: Additional file 1: Figure S1. — SW620 cells were treated with 3c (5 μM) in the absence and presence of NAC (5 mM), Cell viability was measured by MTT assay. The results are expressed as mean of 3 independent determinations (mean ± SD). Figure S2. HT-29 cells were treated with different concentration of Doxorubicin in the absence and presence of 3c (5 μM), Cell viability was measured by MTT assay. The results are expressed as mean of 3 independent determinations (mean ± SD). Figure S3. MCF10A cells were treated with different concentration of 3c, Cell viability was measured by MTT assay. The results are expressed as mean of 3 independent determinations (mean ± SD). Figure S4. SW620 cells treated with 3c were incubated with c-H2DCFDA for 15 min. Fluorescence of oxidized DCF was measured by flow cytometry. Figure S5. 3c treated SW620 cells were incubated with rhodamine 123 and analyzed by flow cytometry. (PPTX 115 kb) [file 12885_2016_3005_MOESM1_ESM.pptx]

## Slide 1
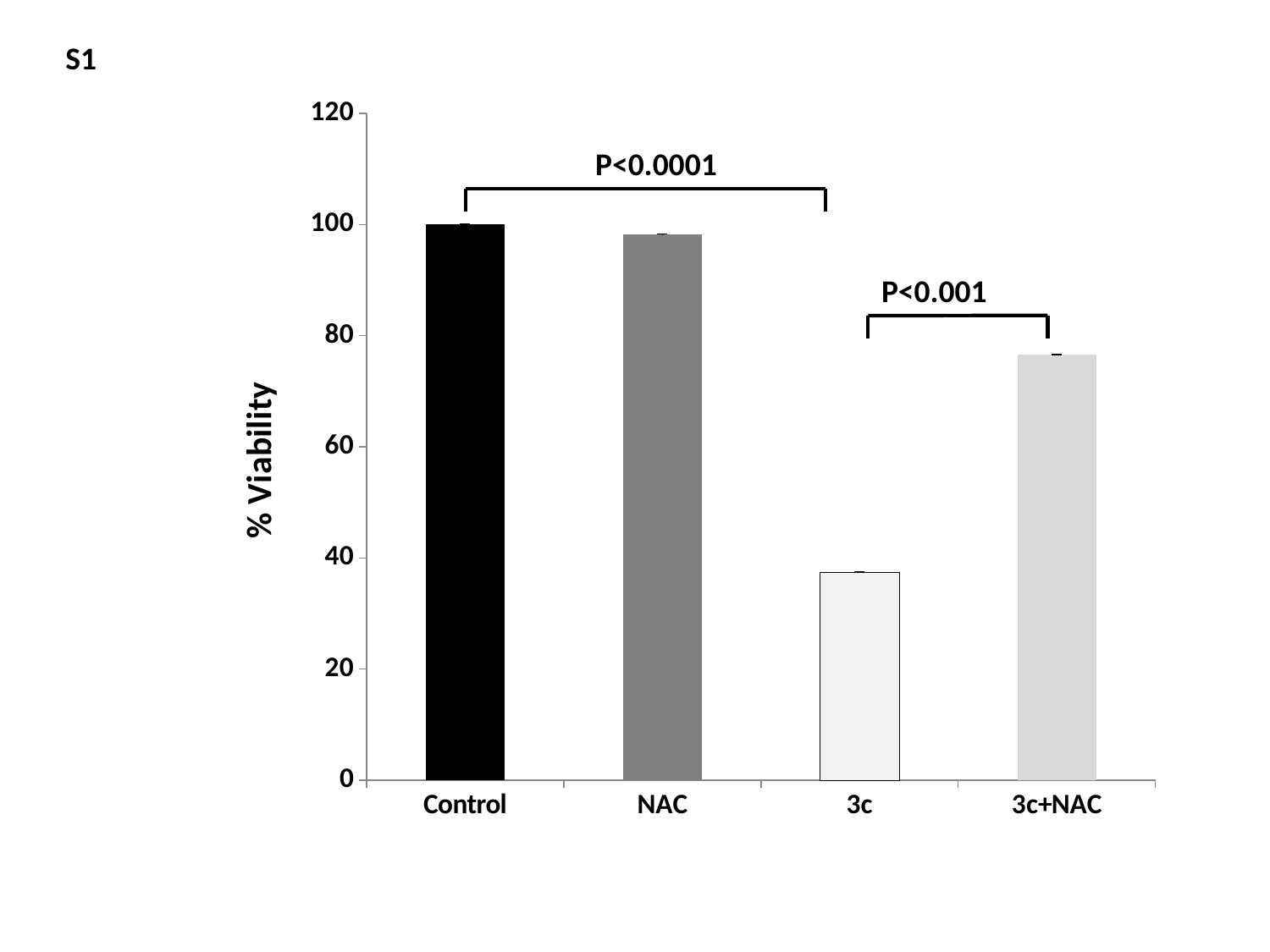

S1
### Chart
| Category | |
|---|---|
| Control | 100.0 |
| NAC | 98.3 |
| 3c | 37.5 |
| 3c+NAC | 76.6 |P<0.0001
P<0.001

## Slide 2
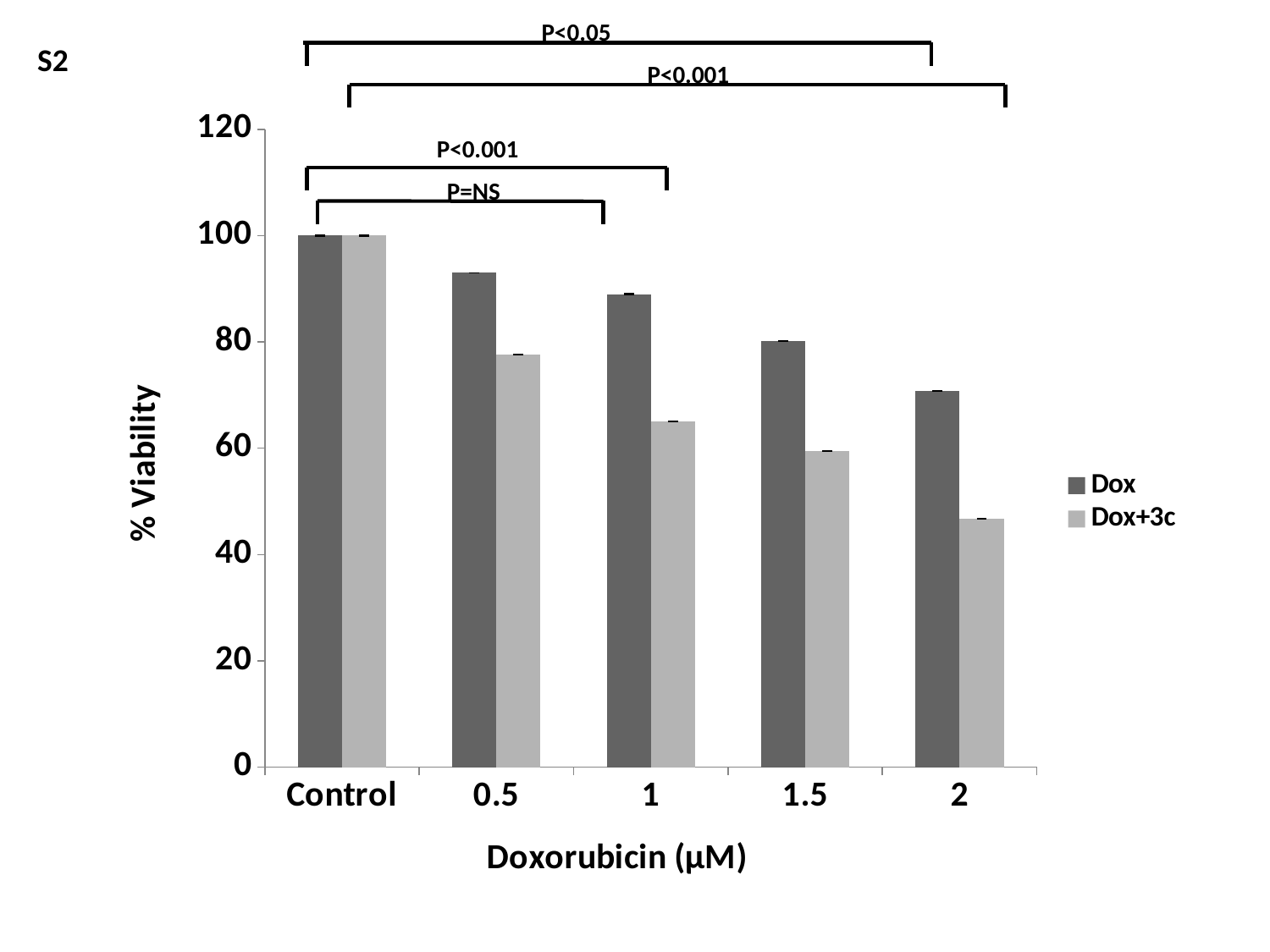

P<0.05
S2
P<0.001
### Chart
| Category | Dox | Dox+3c |
|---|---|---|
| Control | 100.0 | 100.0 |
| 0.5 | 93.0 | 77.6 |
| 1 | 89.0 | 65.0 |
| 1.5 | 80.2 | 59.5 |
| 2 | 70.8 | 46.8 |P<0.001
P=NS

## Slide 3
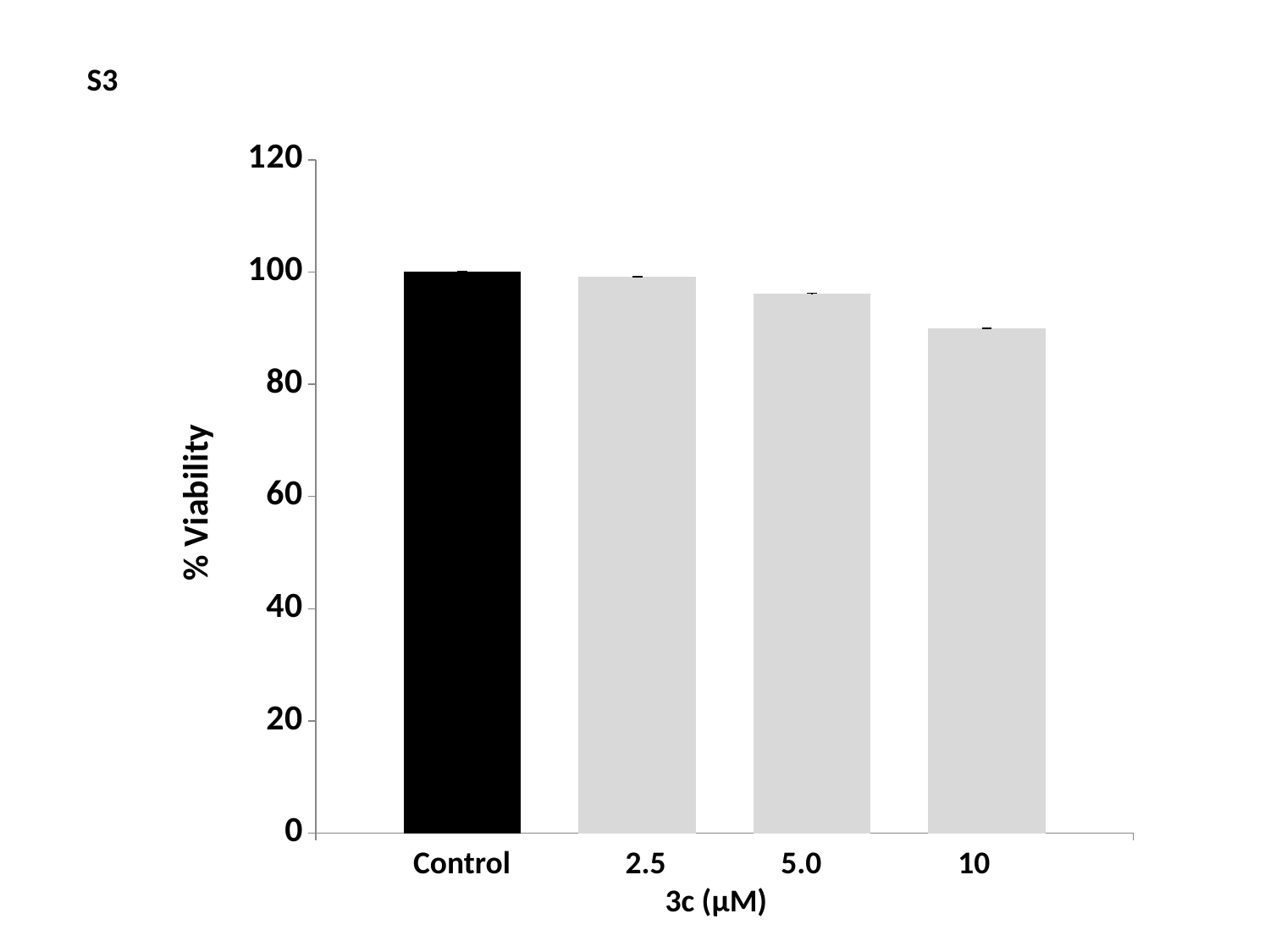

S3
### Chart
| Category | | | | |
|---|---|---|---|---|Control 2.5 5.0 10
 3c (µM)

## Slide 4
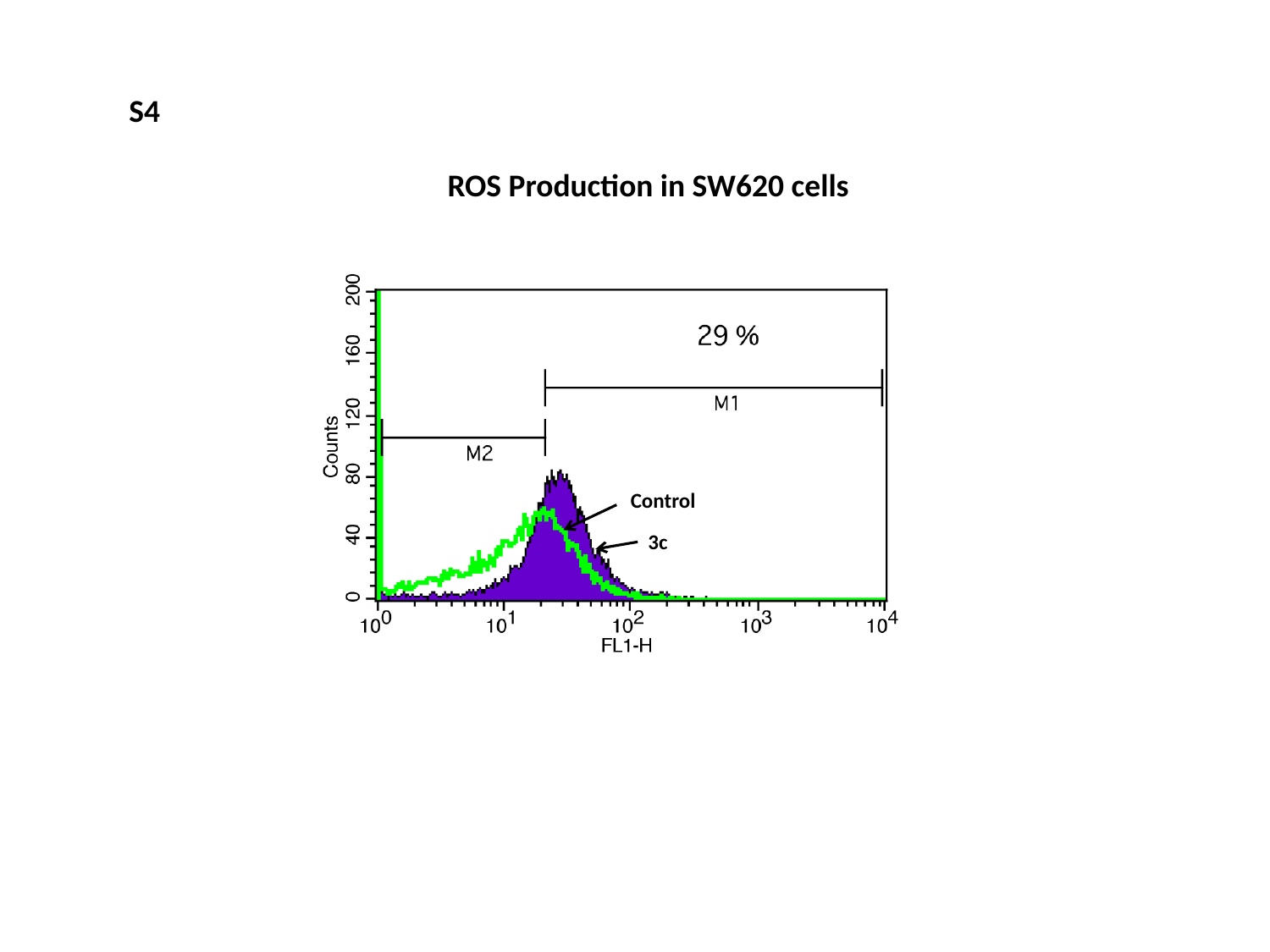

S4
ROS Production in SW620 cells
Control
3c

## Slide 5
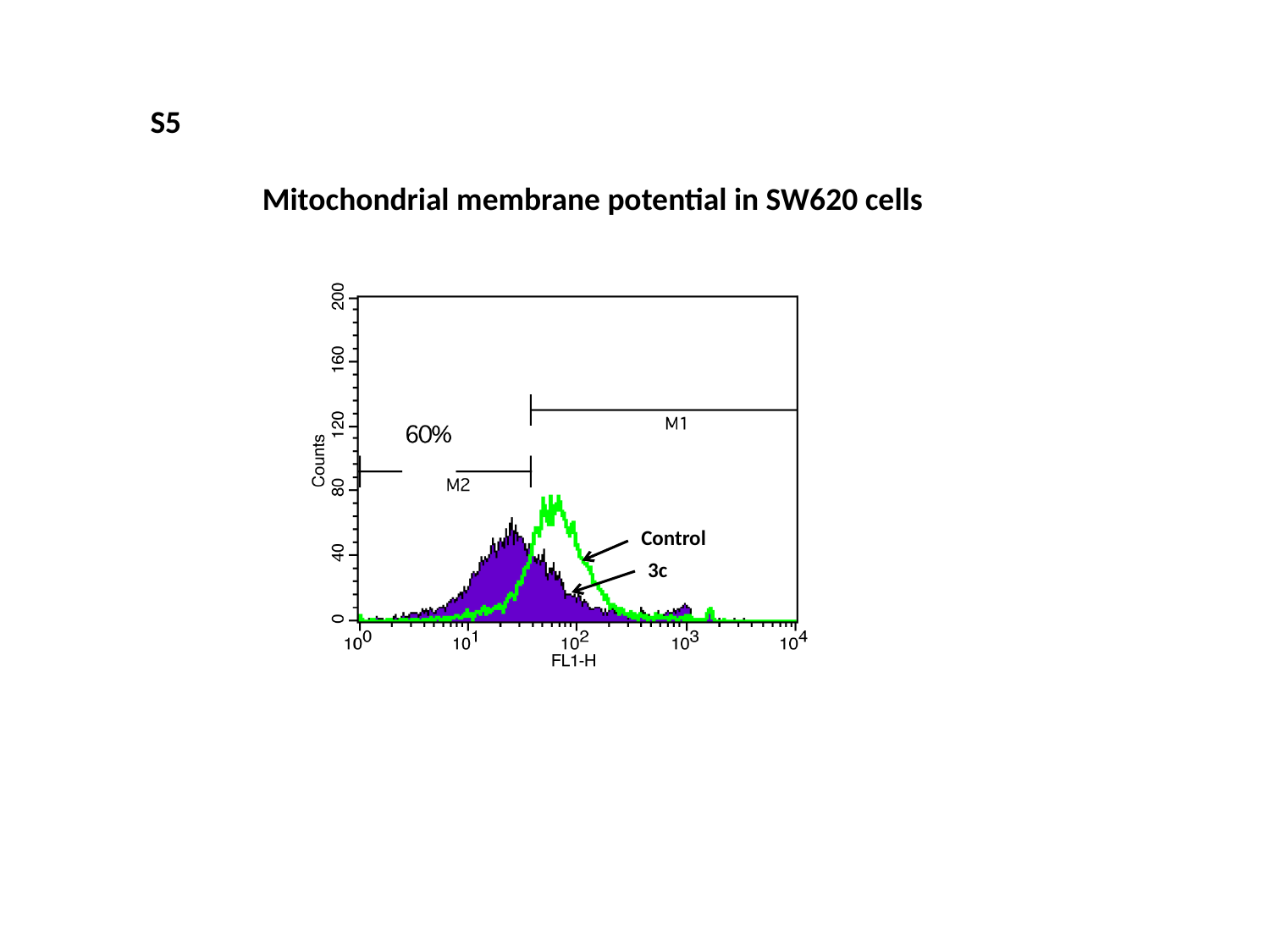

S5
Mitochondrial membrane potential in SW620 cells
Control
3c
